# Melatonin Non-Linearly Modulates Bull Spermatozoa Motility and Physiology in Capacitating and Non-Capacitating Conditions

**DOI:** 10.3390/ijms21082701

**Published:** 2020-04-13

**Authors:** Estela Fernández-Alegre, Indira Álvarez-Fernández, Juan Carlos Domínguez, Adriana Casao, Felipe Martínez-Pastor

**Affiliations:** 1 Institute of Animal Health and Cattle Development (INDEGSAL), University of León, 24071 León, Spain; efernandez@bianorbiotech.es (E.F.-A.); indira.alvarez.fdez@gmail.com (I.Á.-F.); jcdomt@unileon.es (J.C.D.); 2 Department of Animal Medicine, Surgery and Anatomy (Animal Medicine and Surgery), University of León, 24071 León, Spain; 3 BIOFITER, Department of Biochemistry and Molecular and Cell Biology, Institute of Environmental Sciences of Aragón, School of Veterinary Medicine, University of Zaragoza, 50013 Zaragoza, Spain; adriana@unizar.es; 4 Department of Molecular Biology (Cell Biology), University of León, 24071 León, Spain

**Keywords:** melatonin, spermatozoon, bull, sperm motility, sperm physiology

## Abstract

Bull spermatozoa physiology may be modulated by melatonin. We washed ejaculated spermatozoa free of melatonin and incubated them (4 h, 38 °C) with 0-pM, 1-pM, 100-pM, 10-nM and 1-µM melatonin in TALP-HEPES (non-capacitating) and TALP-HEPES-heparin (capacitating). This range of concentrations encompassed the effects mediated by melatonin receptors (pM), intracellular targets (nM–µM) or antioxidant activity (µM). Treatment effects were assessed as motility changes by computer-assisted sperm analysis (CASA) of motility and physiological changes by flow cytometry. Melatonin effects were more evident in capacitating conditions, with 100 pM reducing motility and velocity (VCL) while increasing a “slow” subpopulation. All concentrations decreased apoptotic spermatozoa and stimulated mitochondrial activity in viable spermatozoa, with 100 pM–1 µM increasing acrosomal damage, 10 nM–1 µM increasing intracellular calcium and 1 pM reducing the response to a calcium-ionophore challenge. In non-capacitating media, 1 µM increased hyperactivation-related variables and decreased apoptotic spermatozoa; 100 pM–1 µM increased membrane disorders (related to capacitation); all concentrations decreased mitochondrial ROS production. Melatonin concentrations had a modal effect on bull spermatozoa, suggesting a capacitation-modulating role and protective effect at physiological concentrations (pM). Some effects may be of practical use, considering artificial reproductive techniques.

## 1. Introduction

Melatonin is a ubiquitous molecule fundamental for animal physiology. While mainly known for its hormonal activity, transducing photoperiodic cues, it is a multifunctional molecule. Indeed, it shows diverse effects both at tissue and cell levels [1]. Synthesis and activity (through transmembrane or intracellular receptors or as an antioxidant) occur in many tissues and organs [2,3]. Regarding reproduction, it is a key player not only in the seasonal regulation of sexual activity but also locally by regulating the reproductive organs [4,5], where it possibly acts as a paracrine signal as well as an antioxidant [6]. Many reproductive tissues in the male can produce melatonin, as demonstrated in ram [7]. These tissues would be able to respond to locally produced melatonin due to the presence of membrane receptors (MT1 and MT2) [8]. It also accumulates in secretions and other fluids such as the seminal plasma [9,10,11] and the follicular fluid [12], affecting the physiology of the spermatozoon both within the male and the female.

Indeed, melatonin promotes sperm viability and modifies its physiology, as confirmed in different species [13,14,15,16]. Notably, it appears to modulate capacitation [17]. This is a process occurring in the oviduct, enabling the spermatozoa to fertilize the oocyte. Capacitation starts by membrane modifications resulting in changes in the intracellular concentrations of second messengers and other mediators such as cAMP, ROS and calcium, activating signal transduction cascades [18,19]. Melatonin could affect the spermatozoon at different levels; previous studies have found a variety of effects, sometimes contrary, in different species and concentrations. Some protective and anti-apoptotic effects could be attributed by its antioxidant activity at the micro or millimolar levels, either by direct removal of oxidant species or indirectly by promoting survival signaling pathways [13,14,16]. Moreover, melatonin may have a chemotactic effect during capacitation, contributing to sperm guidance towards the fertilization site [4].

At lower concentrations, melatonin seems to modulate capacitation and related events, such as hyperactivation [14,15,17]. Melatonin likely exerts such activities by binding its membrane receptors MT1 and MT2, which have been detected in the spermatozoon of many species [10,20]. These receptors are high-affinity binding sites for melatonin, but there are other putative intracellular targets considered as low-affinity binding sites [21,22]. This is further complicated by the tendency of the membrane receptors to homo or heterodimerize, which may modify both their affinity to melatonin and the kind of transduced signal [21,23]. In other cell types, the activation of these receptors (members of the G protein-coupled receptors family, GPCRs) reduces adenyl cyclase activity through inhibitory G proteins, thus reducing intracellular cAMP and PKA activity [21,24]. Recently, Gimeno-Martos et al. (2018) [17] demonstrated an effect of melatonin at micromolar concentrations on sperm capacitation through modulation of cAMP levels. This observation is very relevant, since the activation of PKA in sperm capacitation, mediated by a fast cAMP increase and the subsequent phosphorylation of many intracellular targets is a significant event [25,26,27]. Whereas nano or picomolar melatonin concentrations did not present that effect, sperm motility was affected at these levels, at least in a subpopulation of spermatozoa [17].

Our objective in the present study is to clarify the effect of melatonin at different concentrations in the physiology of bull spermatozoa. There is a lack of information on this important farm species, with only a few reports focusing on the antioxidant protection of melatonin in the millimolar-nanomolar range [16,28]. Reports on melatonin in the picomolar range—where only membrane receptors should mediate its effects—are still scarce regarding mammal spermatozoa. Except for some studies in hamster [15] and sheep [14,17], most experiments have tested melatonin at concentrations which confound effects mediated by membrane receptors with those due to its antioxidant activity or mediated by putative intracellular targets [1,29,30].

We also hypothesize that bull spermatozoa would respond differently to melatonin at capacitating and non-capacitating conditions, as found in sheep [17]. Spermatozoa find different melatonin concentrations in the male genital tract, seminal plasma and the different regions of the female genital tract; that is, in non-capacitating and capacitating environments [4,10,31,32]. There is a lack of systematic research on these topics, which would help us to understand sperm physiology better. Whereas many studies have focused on the effects on hyperactivation and capacitation-inducing conditions [15,16,17], they are scarce regarding non-capacitating conditions while using melatonin within the physiological range [14,17]. Moreover, this research would contribute not only with basic knowledge but also with practical applications in the field of animal breeding. Melatonin has the potential for improving IVF and other assisted reproductive techniques involving capacitating treatments [28,33]. However, melatonin is also very interesting as a supplement for sperm storage media [34,35,36]. In these conditions, spermatozoa must remain as quiescent as possible. We could take advantage of concentrations in which melatonin would prevent, rather than promote, capacitation while taking advantage of its protective properties. Some studies have tested melatonin with this purpose in the millimolar and micromolar range [35,37,38,39]. However, the use of potent antioxidants could have detrimental effects on sperm physiology at these concentrations, making desirable testing physiological concentrations [40]. Indeed, melatonin at one µM decreased progressive motility in ram spermatozoa, maybe due to ROS depletion [17]. These authors also found a different effect at lower concentrations in capacitating and non-capacitating media (reducing capacitation in the former, whereas promoting it in the latter). Due to the practical importance of such findings, these results should be tested in other species.

In this study, we have analyzed a wide range of sperm quality variables to assess melatonin effects. Ultimately, the purpose of any reproductive technique is to achieve the oocyte fertilization, resulting in a viable embryo and a successful pregnancy with healthy offspring. The spermatozoon must accomplish many requirements regarding maturity, motility, viability and the ability to respond to external stimuli (especially in the female reproductive tract) and to interact and fertilize the oocyte [41]. Therefore, we included several objective sperm quality analyses, comprising computer-assisted sperm analysis (CASA) of the motility [42] and flow cytometry assessment of sperm physiology [43]. These techniques yield individual information from each cell, enabling carrying out sperm subpopulation analyses. Thus, regarding CASA analysis, we also identified different sperm subpopulation with varying patterns of motility [44,45,46], to get a better insight of melatonin and media effects on the sperm physiology.

Therefore, we have applied a wide range of melatonin concentrations, from one pM to one µM, to clarify the effects on bull spermatozoa at concentrations that could be found in seminal plasma (high to low picomolar [10]) or the oviduct (as a gradient from the ovulating follicle [4,47]). With this purpose, we evaluated the changes on the motility pattern, viability, capacitation, mitochondrial activity, intracellular levels of reactive oxygen species (ROS) and other physiological variables, after incubating bull spermatozoa both in capacitating and non-capacitating conditions.

## 2. Results

Figure 1, Figure 2 and Figure 3 display the average values of the variables considered in this study in the initial (after the sucrose cushion centrifugation) assessment and after the incubations (control tubes), for reference. The Supplementary Material contains the effect sizes of melatonin concentrations within each incubation type (Figures S1–S5), which are displayed as mean ± SEM of the respective treatments in Figure 4, Figure 5, Figure 6, Figure 7 and Figure 8.

### 2.1. Effects of Melatonin Concentrations on Sperm Motility

The melatonin concentrations showed little effect on sperm motility as assessed by CASA (Figure 4). In non-capacitated samples, incubation with 1 µM significantly increased some hyperactivation-related variables: ALH (lateral movement of the head, Figure 4i), DNC and DNCm (*p* < 0.05, Figure 4k,l). However, we did not observe a concomitant decrease in sperm linearity variables (LIN or STR). Considering the capacitating conditions, 100 nM depressed motility respect to the control, exerting a significant impact on total motility, progressive motility, sperm velocity as VCL and BCF (Figure 4c,j).

The cluster analysis yielded three subpopulations (Table 1), named according to their average kinematic values. The “Active” cluster grouped the fastest spermatozoa (being also the predominant one in all cases; Figure 5a). Whereas these spermatozoa showed mostly linear trajectories (median STR > 80%), their flagellar beat was very vigorous, with a broad lateral head movement (ALH) and consequently the highest DNC and DNCm. The “Rapid” cluster grouped those with relatively high velocity and linearity, but less active flagellar beating (BCF). The “Slow” cluster grouped those with the slowest and most erratic swimming. The proportions of the Active cluster were not significantly affected by the melatonin incubation (Figure 5a). However, the incubation in capacitating conditions with 100-pM melatonin had a significant decreasing effect on Rapid (Figure 5b) and an increasing concomitant effect on Slow (Figure 5c).

### 2.2. Effects of Melatonin Concentrations on Sperm Physiology (Flow Cytometry Analyses)

The analysis of sperm physiology by flow cytometry (Figure 6) revealed different dynamics between the two incubation groups. The samples incubated in the non-capacitating medium showed a significant small decrease on the occurrence of apoptosis-like changes at 1 µM (Figure 6b) and an increase in membrane disorder (associated to sperm capacitation) at 100 pM–1 µM (Figure 6e). All melatonin concentrations significantly reduced the production of mitochondrial superoxide in viable spermatozoa (Figure 6i), with a stronger effect for the 10-nM and1-µM concentrations.

**Figure 2:**
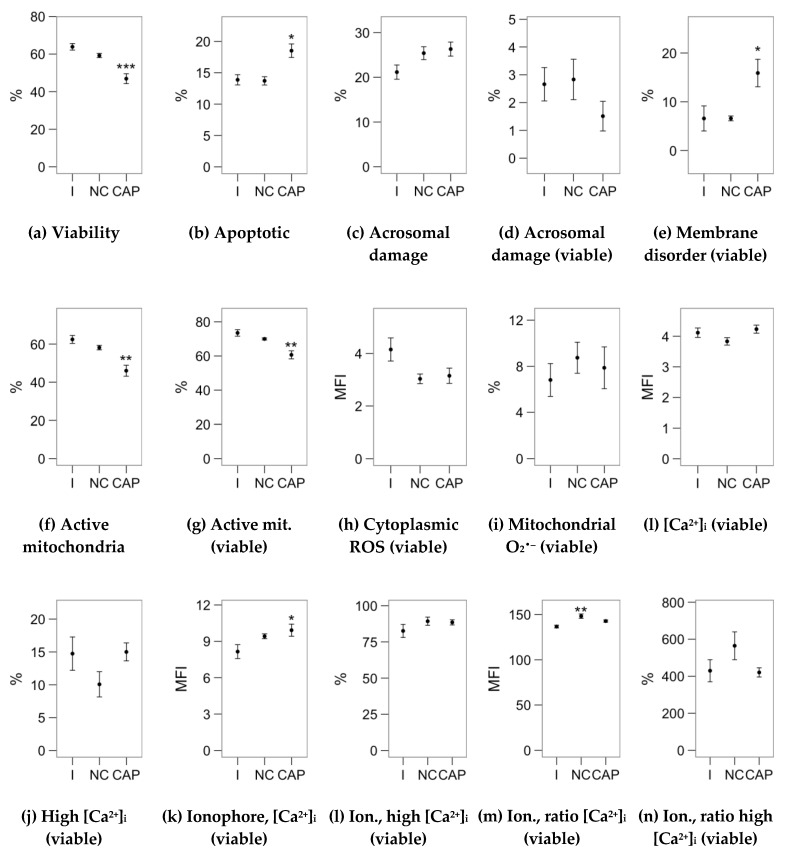
Results of sperm analyses (sperm physiology by flow cytometry) in the unprocessed samples (initial assessment, I) and after the incubation (4 h at 37 °C and 5% CO_2_) in non-capacitating (NCAP) and capacitating (CAP, 2 UI/mL heparin) conditions. Plots show mean s± SEM. (**a**) Viable spermatozoa. (**b**) Apoptotic spermatozoa (YO-PRO-1^+^). (**c**) Acrosome-reacted (damaged) spermatozoa. (**d**) Spermatozoa with damaged acrosome as the ratio of viable spermatozoa. (**e**) Capacitated spermatozoa as with increased membrane disorder (M540+), as the ratio of viable spermatozoa. (**f**) Spermatozoa with active mitochondria. (**g**) Spermatozoa with active mitochondria as the ratio of viable spermatozoa. (**h**) Cytoplasmic ROS production of viable spermatozoa. (**i**) Spermatozoa with high mitochondrial superoxide production as the ratio of viable spermatozoa. (**j**) Intracellular calcium concentration ([Ca^2+^]_i,_ from mean fluorescence intensity of Fluo-4 in viable spermatozoa). (**k**) Spermatozoa with high [Ca^2+^]_i_ as the ratio of viable spermatozoa. (**k,l**) Same parameters after ionophore treatment. (**m,n**) Same parameters as the ratio of measurements after and before the ionophore treatment (ratio of change). Asterisks indicate significant differences of the incubated samples with the initial assessment (* *p* < 0.05, ** *p* < 0.01, *** *p* < 0.001).

**Figure 4:**
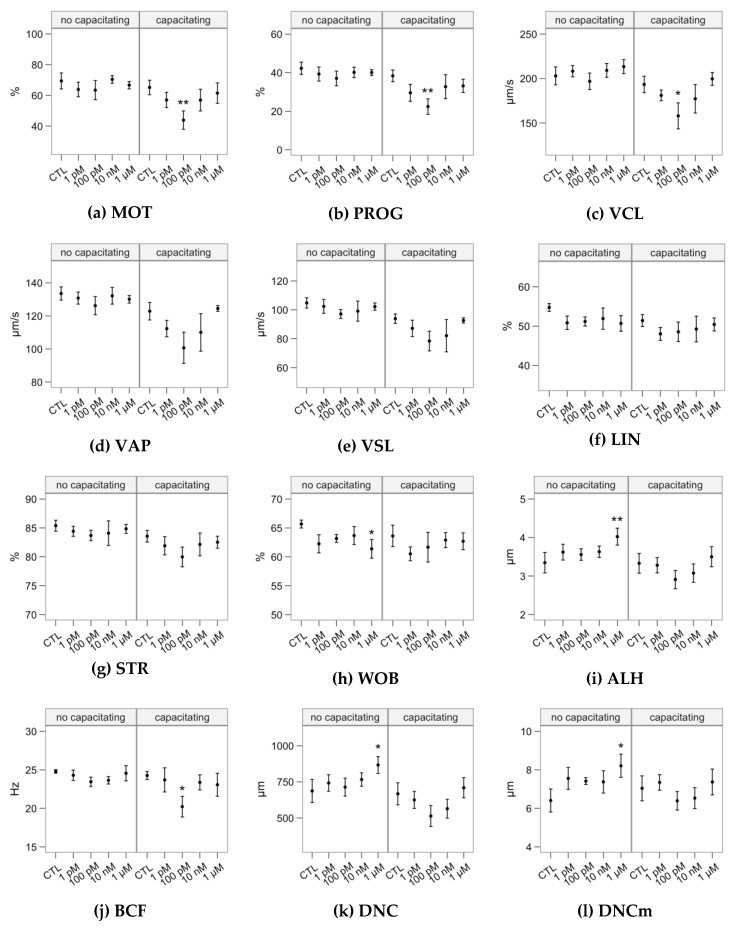
Effects of incubating bull spermatozoa with different melatonin concentrations on motility parameters (CASA analysis; Control, no melatonin, as reference). Plots show mean ± SEM for each concentration in the two in the absence (no capacitating) or presence (capacitating) of heparin (variable names expanded in Figure 1). Asterisks label melatonin effects significantly different from the Control (* *p* < 0.05, ** *p* < 0.01).

**Figure 5:**
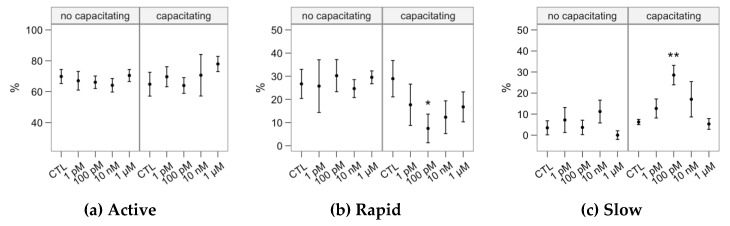
Effects of incubating bull spermatozoa with different melatonin concentrations on motility parameters (CASA analysis; Control, no melatonin, as reference). Plots show mean ± SEM for each concentration in the two in the absence (no capacitating) or presence (capacitating) of heparin (variable names expanded in Figure 1). Asterisks label melatonin effects significantly different from the Control (* *p* < 0.05, ** *p* < 0.01).

**Figure 6:**
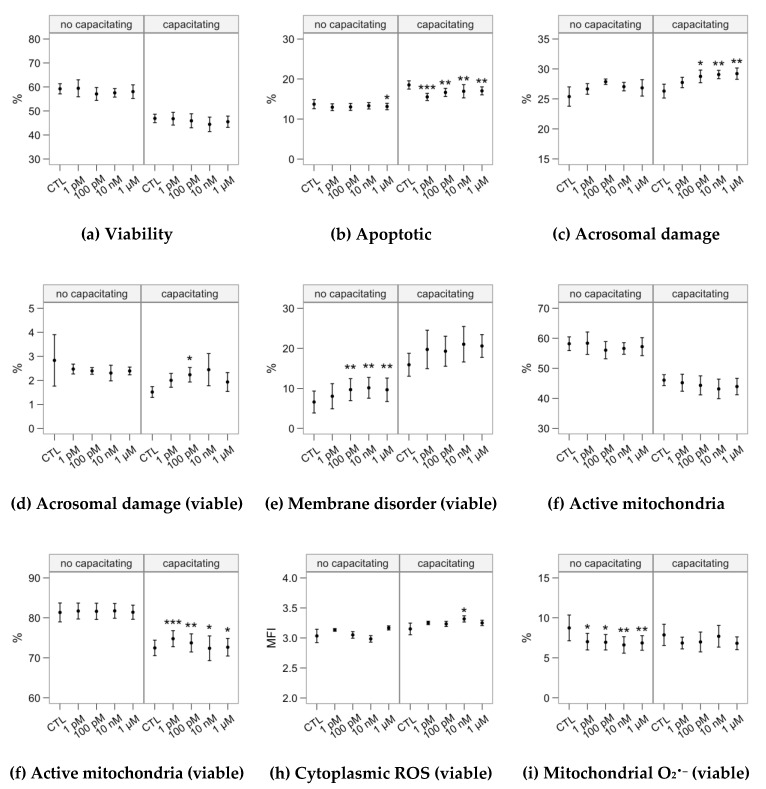
Effects of incubating bull spermatozoa with different melatonin concentrations on some physiological parameters (flow cytometry analysis; Control, no melatonin, as reference). Plots show mean ± SEM for each concentration in the two in the absence (no capacitating) or presence (capacitating) of heparin (variable names expanded in Figure 2). Asterisks label melatonin effects significantly different from the Control (* *p* < 0.05, ** *p* < 0.01, *** *p* < 0.001).

When heparin was included in the incubation medium, we observed a significant effect causing a decrease on the occurrence of apoptosis-like changes (Figure 6b) and an increase on mitochondrial activity (as the ratio of viable spermatozoa; Figure 6f) in all the melatonin concentrations, with a more substantial effect for 1 pM. This is especially relevant considering that the capacitating incubation increased the proportion of apoptotic-like spermatozoa while decreasing the proportion of spermatozoa with active mitochondria (Figure 2b,f,g). The 100 pM–1 µM concentrations significantly promoted acrosomal damage as PNA^+^ events (Figure 5c). However, when considering only viable spermatozoa, only the effect of 100 pM was significant (Figure 6d).

### 2.3. Effects of Melatonin Concentrations on Intracellular Calcium Concentration and the Response to the Ionophore Challenge (Flow Cytometry Analyses)

**Figure 7:**
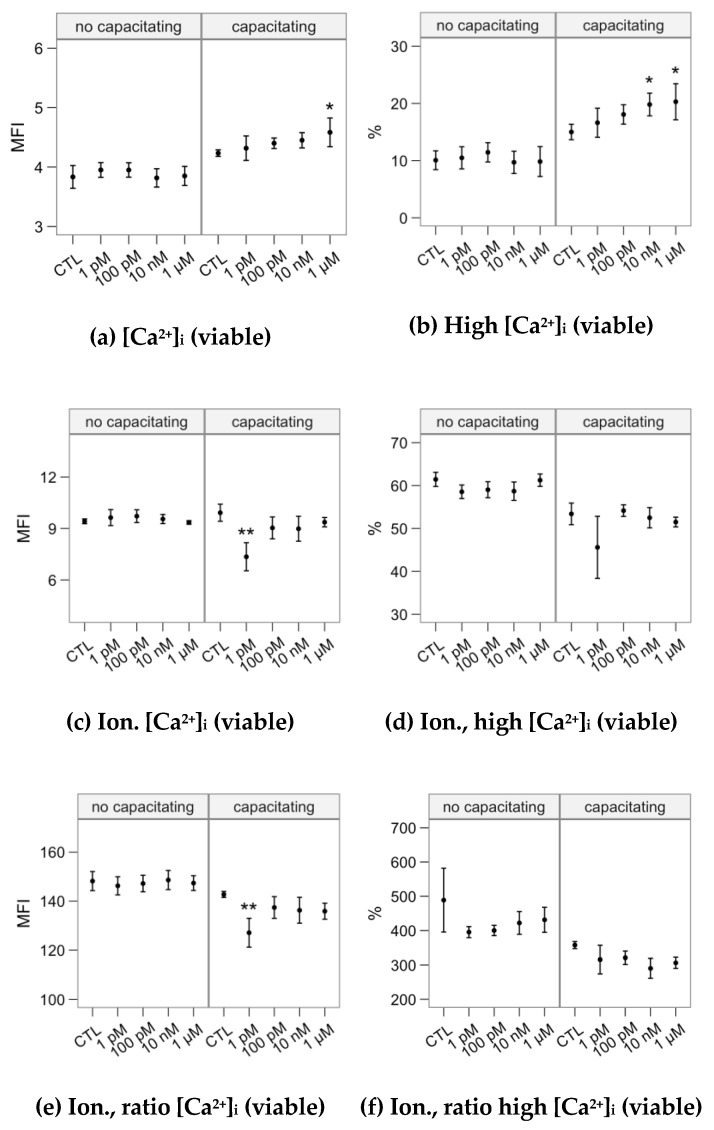
Effects of incubating bull spermatozoa with different melatonin concentrations on intracellular calcium concentration (Control, no melatonin, as reference). Plots show mean ± SEM for each concentration in the two in the absence (no capacitating) or presence (capacitating) of heparin. (variable names expanded in Figure 2). Asterisks label melatonin effects significantly different from the Control (** *p* < 0.01).

Melatonin concentrations did not significantly affect intracellular calcium concentration in the non-capacitating incubation (Figure 7), neither for the basal levels nor after the ionophore challenge. In the presence of heparin, the 1 µM concentration showed a significant effect increasing the Fluo-4 fluorescence (Figure 7a) and 10 nM and 1 µM significantly increased the proportion of spermatozoa showing the highest [Ca^2+^]_i_ (Fluo-4 fluorescence; Figure 7b). The 1-pM concentration modulated down the calcium increase after the ionophore challenge (Fluo-4 fluorescence; Figure 7c,e), with no significant effect for the other concentrations.

### 2.4. Effects of Melatonin Concentrations on the Response to Lysophosphatidylcholine Challenge (Flow Cytometry Analyses)

**Figure 8:**
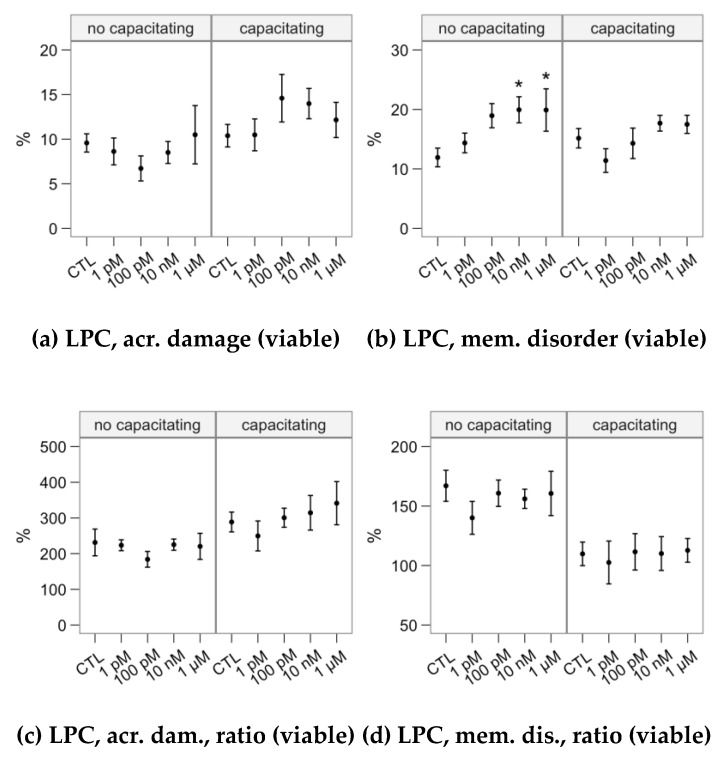
Effects of incubating bull spermatozoa with different melatonin concentrations on the response to the lysophosphatidylcholine challenge (Control, no melatonin, as reference). Plots show mean ± SEM for each concentration in the two in the absence (no capacitating) or presence (capacitating) of heparin (variable names expanded in Figure 3). The melatonin treatment did not show a significant effect on these variables.

We studied the response to the lysophosphatidylcholine (LPC) challenge (Figure 8) as the change on acrosomal damage (PNA^+^ events) and membrane disorder (M540^+^ for viable spermatozoa). The 10-nM and 1-µM melatonin concentrations in the non-capacitating incubation significantly increased the proportion of viable spermatozoa with altered membrane fluidity (Figure 8b). However, the ratio of change was not affected (Figure 8d), due to a similar effect in non-challenged samples (Figure 6e). The LPC-challenge response was not significantly affected by incubating the samples with melatonin in capacitating conditions.

## 3. Discussion

We have provided evidence that melatonin, even at the low 1-pM concentration, affects the physiology of bull spermatozoa. Whereas the effect of melatonin on sperm biology has drawn increasing attention [4,10,14,15,16,32,34,48,49], there is still little information on its relevance regarding this cell type. Most studies have focused on its practical application in artificial reproductive techniques and on its effects as an antioxidant [35,39,50,51,52], mostly using concentrations well above the nanomolar range. These concentrations confound the effects of melatonin as a ligand for membrane receptors (affinity by the picomolar), for intracellular targets (quinone reductase 2, calmodulin, etc., with affinity by the nanomolar) or as an antioxidant (when used from micro to millimolar) [1,29].

In our experiments, we first separated the spermatozoa from the seminal plasma, following studies on ram semen [17,32], as it contains a significant amount of melatonin [10] synthetized in the male genital tract [7]. Bull semen also contains melatonin [10]; we have confirmed the presence of key enzymes in melatonin synthesis in many tissues of the bull genital tract (unpublished). However, we must consider that the samples remained in contact with seminal plasma for at least 30 min after collection. Therefore, the exposure to the endogenous melatonin could have been relevant. Melatonin receptors could undergo relocation or internalization upon melatonin binding, as suggested recently for ram [32] and bull (unpublished results by the authors).

Our results show that melatonin differentially affects bull spermatozoa depending on incubation conditions. Previous studies in the sheep model (possibly the most detailed up to date) indicate that incubation conditions could affect melatonin response [14,17,32]. We have to highlight that many experiments in ram used a capacitating cocktail combining sperm stimulants and inhibitors of phosphodiesterase and phosphatases. This complex combination is necessary because the ram spermatozoon is difficult to capacitate in vitro [53]. In contrast, heparin (in a standard medium with calcium and bicarbonate) is an efficient capacitation inducer for bull spermatozoa [54]. Thus, we could capacitate bull spermatozoa with this simple system. We found an inhibitory effect of 100-pM melatonin on sperm motility only in these capacitating conditions, however not associated with a decrease in sperm viability. This motility inhibition also reflected on the subpopulation distribution, with part of the spermatozoa significantly shifting from the “Rapid” to the “Slow” cluster. Fujinoki [15] describes the effects of melatonin on hamster sperm motility (1 pM to 1 µM), but in this case, as a strong promoter of sperm hyperactivation. However, there are ample differences between species, and other studies did not report that dramatic effect. Melatonin stimulated sperm motility at millimolar in human [31], with diverse outcomes in pig spermatozoa when used at either milli or micromolar [48,55].

Experiments in sheep did not found any effect on the motility from 100 pM to 1 µM [14]. Still— using the capacitating cocktail—melatonin promoted an increase in progressivity at 10 nM, and a decrease with 1 µM [17]. Interestingly, a subpopulation with fast and linear spermatozoa increased at 100 pM and 10 nM. This is in contrast with our findings at 100 pM, but somewhat resemble some results in boar [48]. Incubation of boar spermatozoa with melatonin at micromolar concentrations decreased motility and kinematic parameters (indeed, 5-µM abolished motility) unless a stimulant (progesterone) was applied. Sperm motility strongly depends on intracellular Ca^2+^ levels ([Ca^2+^]_i_), and we found that melatonin could modulate [Ca^2+^]_i_ in capacitating conditions (but not in the absence of heparin). However, these effects consisted of an increase of [Ca^2+^]_i_ in the highest melatonin concentrations and a refractory effect against the ionophore challenge at 1 pM. It is possible that these effects are too small for affecting motility in a detectable manner, or that a simultaneous inhibitory effect of melatonin prevents these changes to translate into an increase of the axonemal activity. The calcium changes must turn into the phosphorylation of many proteins in the axoneme to modify the flagellar beating [56]. However, nanomolar melatonin should be able to inhibit calmodulin, a relevant calcium mediator in the flagellum; at higher concentrations, it could even inhibit other pathways possibly related to ROS signaling. Reports on ram spermatozoa hint in that direction, with changes in intracellular calcium distribution (assessed by chlortetracycline) not reflecting in varying sperm motility [14,17].

Li et al. [16] tested the effect of melatonin on sorted bull spermatozoa in the nano–millimolar range, with higher concentrations increasing [Ca^2+^]_i_. (using Fluo-3, a probe similar to Fluo-4), concomitant to a cAMP rise. Interestingly, this increase was more evident in the milli–micromolar range, matching our results in capacitating conditions (higher membrane disorder). These conditions also resulted in a rise of mitochondrial activity, however not observed in the nanomolar range. In contrast, we have reported a stimulatory effect of low melatonin concentrations on mitochondrial activity and a protective effect by preventing the appearance of apoptotic features (possibly related, since the mitochondria are essential in the regulation of sperm apoptosis [36]). Li et al. studied spermatozoa that had been not only sex-sorted but also cryopreserved, therefore having undergone a high level of stress. Indeed, our samples showed minimal levels of cytoplasmic and mitochondrial ROS even after the incubations, contrarily to reported in sex-sorted and cryopreserved samples [16]. Higher melatonin levels could indicate a positive effect because of the vulnerable status of these samples, through a direct or indirect antioxidant effect [6], as shown in previous studies with thawed spermatozoa [39].

Nevertheless, we also observed a generalized decrease in mitochondrial-produced ROS when incubating in non-capacitating conditions with melatonin. Melatonin could accumulate in the mitochondria, exerting an antioxidant and protective activity in this organelle [57]. However, the effects of picomolar and micromolar concentrations were similar; thus, this effect may rather be mediated via the membrane receptors instead. Indeed, the impact of these picomolar concentrations (unlikely to produce an antioxidant effect of to affect intracellular targets significantly) in capacitating conditions strongly advocate for a role for the melatonin membrane receptors in bull sperm physiology. Besides, some results on [Ca^2+^]_i_, namely a lower response to the ionophore challenge, were only observed in samples incubated in capacitating conditions in the presence of 1-pM melatonin.

Effects of melatonin on second messengers such as cAMP were also recently reported in ram spermatozoa [17]. cAMP is a fundamental mediator of many sperm processes, crucially capacitation by promoting the phosphorylation of many proteins by PKA activation [58]. In these studies, melatonin at micromolar concentrations prevented cAMP and ROS increases elicited by the capacitation-inducing cocktail. Casao et al. [14] reported a promoting effect of melatonin at 100 pM on ram sperm capacitation, but an inhibitory effect at 1 µM. Interestingly, we have found that melatonin not only increased membrane modifications in viable spermatozoa (compatible with capacitation) in non-capacitating conditions, but also induced a rise in cytoplasmic ROS in capacitating conditions at 10 nM. Moreover, at 100 pM and above, melatonin was associated with a more substantial proportion of altered acrosomes, compatible with the stimulation of intracellular pathways related to acrosome docking and reaction [59] (concomitant with higher [Ca^2+^]_i_ levels observed at higher concentrations). Nevertheless, viable spermatozoa with reacted acrosomes increased only in samples incubated in capacitating conditions and at 100 pM, supporting a relevant effect in functional spermatozoa possibly through the activation of membrane receptors, resembling some observations in ram [14].

The different effects of melatonin, also reported in other species [14,16,17,48], could be explained by the different intracellular pathways present in sperm compartments. The spermatozoon is a tightly compartmentalized cell and whereas cAMP synthesis depends on transmembrane adenylate cyclases (tmAC) in the head, the soluble form (sAC) is present in the tail [27]. Whereas tmAC activity is affected by membrane-bound G proteins, bicarbonate is the primary modulator for sAC activity [60]. Therefore, melatonin membrane receptors, being GPCRs, could directly influence cAMP dynamics in the sperm head, by activating inhibitory G proteins as described in other cell types [22]. In contrast, the effects of these receptors should be mediated by different pathways in the flagellum, where sAC is the predominant actor. Indeed, melatonin receptors activate many other pathways, not necessarily mediated by cAMP (MEK 1/2, ERK 1/2, PI3K/Akt, etc.) [61], which are also essential for sperm function and especially for capacitation [58,59,62,63].

Moreover, the main target of cAMP is PKA, a potent promoter of protein phosphorylation in spermatozoa [64]. Still, other proteins also respond to this second messenger and seem to have a predominant role in the signaling at the sperm head, such as EPAC (Exchange Protein Directly Activated by cAMP) [65]. Altogether, this could explain some contradictory effects observed in our experiments, such as the depression of motility at low concentrations with the simultaneous prevention of sperm apoptosis (likely, an stimulation of survival pathways as reported in other species [13]) and mitochondrial activation.

Finally, melatonin at the nano or micromolar concentrations could activate not only MT1/MT2 membrane receptors but also directly affect other intracellular targets (calmodulin, quinone reductase 2, etc.) [23,66]. This could add to explaining the modal response in calcium dynamics at different melatonin concentrations, also reported in ram by using chlortetracycline [17]. A further complication is that melatonin could induce receptor relocation or internationalization. There is evidence that MT1 and MT2 receptors present a heterogeneous distribution on the sperm membrane, both depending on the species [10,20] and the capacitation status, as reported in ram [32] and possibly bull and deer (unpublished results). MT2, which seems to be a major player in sperm functionality in ram [32], was found in the sperm neck in the bull; it collocates with MT1 at least in a subpopulation of cells. While still unexplored in spermatozoa, the activation of MT1 or MT2 as homo, dimers or heterodimers results into different intracellular signaling, potentially modifying its final effects on the cell response [21]. A limitation of the present study is that evidence of involvement of MT1 or MT2 was indirect (response to the picomolar concentrations). Follow-up studies should focus on using specific agonists and antagonists of these receptors to confirm their effect on sperm physiology and on relating melatonin exposure to receptor patterns on bull spermatozoa. Reproductive strategies reflect on sperm physiology, which can be very different between species [67]. Thus, future experiments should confirm if the effects of melatonin through membrane receptors can compare to findings in sheep [32].

Our results open new avenues of research, inviting researchers in other species to test wider ranges of melatonin concentrations in both capacitating and non-capacitating conditions. Since melatonin membrane receptors could be ubiquitous in mammal spermatozoa [10], the application of this hormone should go beyond its use as an antioxidant. Melatonin may be applied in a range of applications not only to maintain sperm viability but also to modulate sperm metabolism and physiology. To achieve the objectives of the present study, we limited our experimental setup to a single medium (TALP-HEPES) for the capacitating and non-capacitating conditions. Therefore, we cannot extrapolate our results to other non-capacitating media such as extenders for cooled or cryopreserved storage or to the vast choice of capacitating media for IVF and other ART. Experts may find our results promising for enhancing these media by using melatonin.

## 4. Materials and Methods

### 4.1. Semen Samples and Experimental Design

The semen samples used in this study were provided by an animal breeding center (CENSYRA, León, Spain), which obtained them following routine procedures for producing cryopreserved semen doses from selected bulls. Ejaculates were obtained by artificial vagina from two Limousine and four Holstein bulls (12 to 24 months of age) and kept at 30 °C until processed. An aliquot of the ejaculates was donated to the researchers, who did not interact with the animals. The experimental plan was reviewer by the Animal Care Committee, which cleared it as no concern for animal welfare.

Reagents for solutions and incubations were purchased from Sigma-Aldrich (Merck KGaA, Darmstadt, Germany), unless otherwise stated. Immediately after arrival to the laboratory (within 1 h from collection), the semen was layered on 7,5 mL of a sucrose cushion (10 mM NaCl, 2.5 mM KCl, 20 mM HEPES, 222 mM sucrose, 5-mM glucose and 0.1% PVP; pH 7.3) and centrifuged at 200× *g* for 5 min and at 900× *g* for 10 min. The pellet, free from seminal plasma and debris, was adjusted to 50 × 10^6^ mL^−1^ in TALP-HEPES medium (100 mM NaCl, 3.1 mM KCl, 25 mM NaHCO_3_, 21.6 mM Na lactate, 10 mM HEPES, 5-mM glucose, 3 mM CaCl_2_, 1-mM Na pyruvate, 0.3 mM NaH_2_PO_4_, 0.4 mM MgCl_2_, 2 U/mL gentamicin, 0.5% phenol red and 0.5% BSA; pH 7.3).

The samples were assessed 10 min after the sucrose centrifugation and then split in two tubes, one of them receiving 2 U/mL of heparin (for inducing capacitation). Each tube was split in five tubes, receiving melatonin at 0-pM (control), 1-pM, 100-pM, 10-nM and 1-µM concentrations (0.23 pg/mL to 0.23 µg/mL). The melatonin was prepared in DMSO at 50 mM and then serially diluted so that each tube received 0.2% DMSO (the control tubes received only vehicle).

Analyses were carried out in the original sample (vehicle only) just after preparing the experimental treatments and after incubating the tubes for 4 h at 38 °C, 5% CO_2_.

### 4.2. Sperm Motility Analysis

We used a computer-assisted sperm analysis system (CASA) for analyzing sperm motility. These systems employ image capture and analysis for achieving objective and highly repeatable analysis of sperm motility [42]. The CASA software used for image analysis was ISAS v. 1.19 (Proiser, Valencia, Spain), a research-oriented system which was adjusted for bull spermatozoa as indicated below. An aliquot of sample was extended in TALP-HEPES and observed in a modified Makler chamber (20-µm depth) with a negative phase contrast microscope at 37 °C using negative phase contrast and a ×10 objective. At least 5 fields and 200 cells were recorded using a Basler A312fs digital camera (Basler Vision Technologies, Ahrensburg, Germany) working at 53 fps. The software was adjusted with head area between 25 and 80 µm/s, minimum velocity of 20 µm/s and a search radius of 12 pixels. For each spermatozoon, the CASA provided VCL (curvilinear velocity), VSL (straight path velocity), VAP (average path velocity according to the average smoothed path; µm/s), LIN (linearity), STR (straightness), WOB (wobble), ALH (amplitude of the lateral displacement of the sperm head), BCF (frequency of the flagellar beat), DNC (sperm dance) and DNCm (sperm mean dance). We also obtained total motility (MOT) and progressive motility (PROG, as VCL>25 µm/s and STR > 80%). CASA variables are defined by convention, as described previously [68]. We saved the individual data from each spermatozoon for further processing with R scripts [69], obtaining median parameters for each sample. Subpopulation analysis was performed on the raw data, following a two-step procedure applying the AGNES clustering algorithm [70].

### 4.3. Flow Cytometry Analysis of Sperm Physiology

Fluorescence probes were purchased from ThermoFisher Scientific (Waltham, MA, USA). We used several fluorescence probes combinations to test sperm physiology and their responsiveness to several stimuli. The probes and their final concentrations were: Hoechst 33342 (H342, debris discrimination) at 5 µM; YO-PRO-1 (YP1, apoptotic changes in the plasmalemma) at 100 nM; Fluo-4 (intracellular Ca^2+^ concentration assessment) at 100 nM; CM-H_2_DCFDA (CFDA, cytoplasmic ROS detection) at 5 µM; merocyanine 540 (M540, capacitation-like plasmalemma changes) at 2 µM; propidium iodide (PI, viability) at 1 µM; PNA-Alexa Fluor 647 (PNA, peanut agglutinin, acrosomal status) at 1 µg/mL; MitoTracker deep red (MT, mitochondrial activity) at 100 nM; MitoSOX (MSX, mitochondrial superoxide production) at 1 µM. These probes have been described and used by our group previously [71,72,73]. The probes were combined in TALP-HEPES as H342/Fluo-4/M540/PI/PNA, H342/CFDA/PI, H342/YP/MSX/MT. Spermatozoa were added to each probe combination at 10^6^ mL^−1^ and then incubated for 15 min in the dark and 37 °C before running the samples by a CyAn ADP flow cytometer (Beckman Coulter), suited with diode lasers (405 nm, 488 nm, 635 nm). Flow cytometry solutions were purchased from Beckman Coulter (Brea, CA, USA). We used a 450/50 filter (H342) in the 405 nm line; 530/40 filters (YO-PRO-1, Fluo-4, H_2_DCFDA), 575/25 filters (M540) and 613/20 filters (PI, MitoSOX) in the 488 nm line; and a 665/20 filter (PNA-Alexa Fluor 647, MitoTracker deep red) in the 633 nm line. The acquisition was controlled with the Summit V4.3.02 software. Cytometry data were saved as FCS v.3 files and analyzed with the Weasel v. 3.5 software (Frank Battye, Melbourne, Australia). We obtained the following variables as %: viability as YP^−^PI^−^; Acrosomal damage as PNA^+^; acrosomal damage ratio as PNA^+^ in the YP^−^ population; apoptosis ratio as YP^+^ in the PI^−^ population; capacitation ratio as M540^+^ in the PI^−^ population; mitochondrial activity as MT^+^/YP^−^ spermatozoa and superoxide production ratio as MSX^+^ in YP^−^ population. Mean fluorescence intensity (MFI) was used instead of % of cells for estimating intracellular Ca^2+^ concentration ([Ca^2+^]_i_) and cytoplasmic ROS presence, Fluo-4 and CFDA, respectively, after gating out the PI^+^ population.

Ca^2+^ uptake was induced by pretreating spermatozoa, with the calcium ionophore A23187 (3 µM, Sigma-Aldrich) for 10 min, and then reading the Fluo-4 fluorescence. Thus, we obtained the ratio of increase by using the Fluo-4 measurements with and without ionophore pretreatment. We also tested the susceptibility to induce the acrosome reaction after pretreatment of the samples with lysophosphatidylcholine (LPC), 0.3 mg/mL LPC for 10 min at 37 °C. The samples were re-tested with the H342/Fluo-4/M540/PI/PNA combination after both challenges.

### 4.4. Statistical Analysis

Data were analyzed in the R statistical environment [69] by linear mixed-effects models [74] with the melatonin treatment and incubation type as fixed effects and the sample as the grouping factor of the random part of the model. Ten different semen samples were used in the Results were expressed as estimates (mean ± SEM) of effect sizes of melatonin concentrations within each incubation type. The lmerTest package [75] was used to extract the *p* values for the effect sizes of the treatments respect to the control, establishing a signification threshold at *p* < 0.05.

## 5. Conclusions

We have observed very different responses to melatonin on bull spermatozoa, depending not only on concentration but also on the incubation conditions. Melatonin effects were more evident in capacitating conditions. However, results in non-capacitating conditions could be of interest for understanding the role of melatonin in the male genital tract. Whereas low melatonin levels inhibited capacitation in capacitating conditions, they seemed to have an activating role in non-capacitating media. These results at picomolar levels indicate a modulatory effect potentially related to the activity of high-affinity melatonin membrane receptors, in line with observations in ram spermatozoa. Our findings have a practical impact since they hint that melatonin could improve sperm storage or artificial reproductive techniques, by using it at different levels for modulating specific sperm parameters.

## Figures and Tables

**Figure 1:**
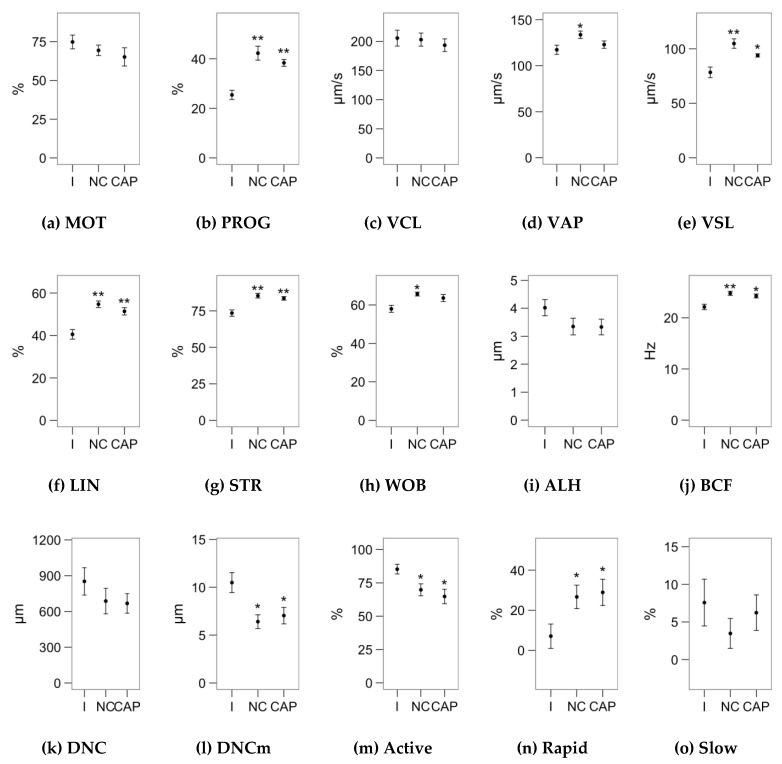
Results of sperm analyses (motility by computer-assisted sperm analysis (CASA) and motility subpopulations) in the unprocessed samples (initial assessment, I) and after the incubation (4 h at 37 °C and 5% CO_2_) in non-capacitating (NCAP) and capacitating (CAP, 2 UI/mL heparin) conditions. Plots show mean s± SEM for each variable and sampling point. (**a**) Total motility. (**b**) Progressive motility. (**c**) Curvilinear velocity. (**d**) Average-path velocity. (**e**) Straight-path velocity. (**f**) Linearity. (**g**) Straightness. (**h**) Wobble. (**i**) Amplitude of the lateral displacement of the sperm head. (**j**) Frequency of the flagellar beat. (**k**) Dance. (**l**) Dance mean. (**m**) “Active” spermatozoa, highest velocity and dance. (**n**) “Rapid” spermatozoa, high velocity and low dance. (**o**) “Slow” spermatozoa, lowest velocity and linearity. Asterisks indicate significant differences of the incubated samples with the initial assessment (* *p* < 0.05, ** *p* < 0.01).

**Figure 3:**
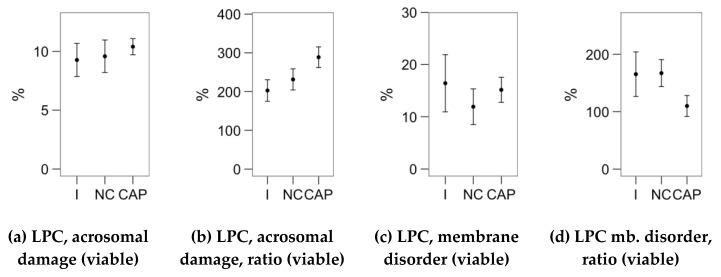
Results of sperm analyses (calcium levels and ionophore and lysophosphatidylcholine–LPC challenges; flow cytometry) in the unprocessed samples (initial assessment, I) and after the incubation (4 h at 37 °C and 5% CO_2_) in non-capacitating (NCAP) and capacitating (CAP, 2 UI/mL heparin) conditions. Plots show mean s± SEM. (**a**) Spermatozoa with damaged acrosome as the ratio of viable spermatozoa. (**b**) Capacitated spermatozoa as with increased membrane disorder (M540+), as the ratio of viable spermatozoa. (**c**,**d**) Same parameters as the ratio of measurements after and before the ionophore treatment (ratio of change). No significant differences of the incubated samples with the initial assessment were detected.

**Table 1:** Descriptive statistics of each sperm cluster obtained after the sperm subpopulation analysis from CASA data. Clusters are described by the median ± SD of the CASA variables, including the overall proportion (all data pooled). VCL: Curvilinear velocity; VSL: Straight path velocity; VAP: Average path velocity according to the average smoothed path; µm/s; LIN: Linearity; STR: Straightness; WOB: wobble; ALH: amplitude of the lateral displacement of the sperm head; BCF: Frequency of the flagellar beat; DNC: Sperm dance; DNCm: Sperm mean dance.

Cluster	VCL	VSL	VAP	LIN	STR	WOB	ALH	BCF	DNC	DNCm	%
Active	218.2 ± 55.1	97.9 ± 43.6	131.8 ± 29.8	48.0 ± 17.0	81.0 ± 16.1	61.5 ± 10.6	4.1 ± 1.5	25.5 ± 6.8	885.8 ± 523.5	8.9 ± 5.8	71.2
Rapid	94.4 ± 38.1	42.2 ± 34.0	61.5 ± 31.1	46.2 ± 24.8	74.8 ± 22.5	64.4 ± 15.4	1.9 ± 0.7	14.9 ± 10.8	175.5 ± 114.4	4.3 ± 3.0	20.5
Slow	50.3 ± 21.0	11.2 ± 8.0	26.2 ± 11.1	24.7 ± 16.8	49.7 ± 25.6	52.8 ± 14.7	1.5 ± 0.5	7.0 ± 4.9	74.5 ± 51.4	6.2 ± 5.0	8.3

## Supplementary Materials

Supplementary materials can be found at https://www.mdpi.com/1422-0067/21/8/2701/s1.

## Abbreviations

ALH	Amplitude of the lateral displacement of the sperm head (µm)
BCF	Frequency of the flagellar beat (Hz)
CM-H_2_DCFDA	Chloromethyl dihydroxy 2′,7′-di-chlorofluorescein acetate
DFI	DNA fragmentation index
%DFI	Proportion of spermatozoa with DFI above threshold (250)
DNC	Sperm dance (VCL × ALH; µm^2^/s)
DNCm	Sperm mean dance (VCL × ALH/VSL; µm)
DMSO	Dimethyl sulfoxide
%HDS	Proportion of spermatozoa with high DNA stainability
LIN	Linearity (VCL/VSL)
LPC	Lysophosphatidylcholine
MOT	Total motility
PROG	Progressive motility
PVP	Polyvinylpyrrolidone
VSL	Straight path velocity (µm/s)
SCSA	Sperm chromatin structure assay
STR	Straightness (VAP/VSL)
TALP	Tyrode-albumin-lactate-pyruvate medium
VAP	Average path velocity (µm/s)
VCL	Curvilinear velocity (µm/s)
WOB	Wobble (VAP/VCL)
