# Supplementary material for: Melatonin Non-Linearly Modulates Bull Spermatozoa Motility and Physiology in Capacitating and Non-Capacitating Conditions
Source: Int J Mol Sci. 2020 Apr 13;21(8):2701. doi: 10.3390/ijms21082701 (PMC7215461; doi:10.3390/ijms21082701)
Supplement: Supplementary file 1 [file ijms-21-02701-s001.pdf]

International Journal of *Molecular Sciences*

MDPI

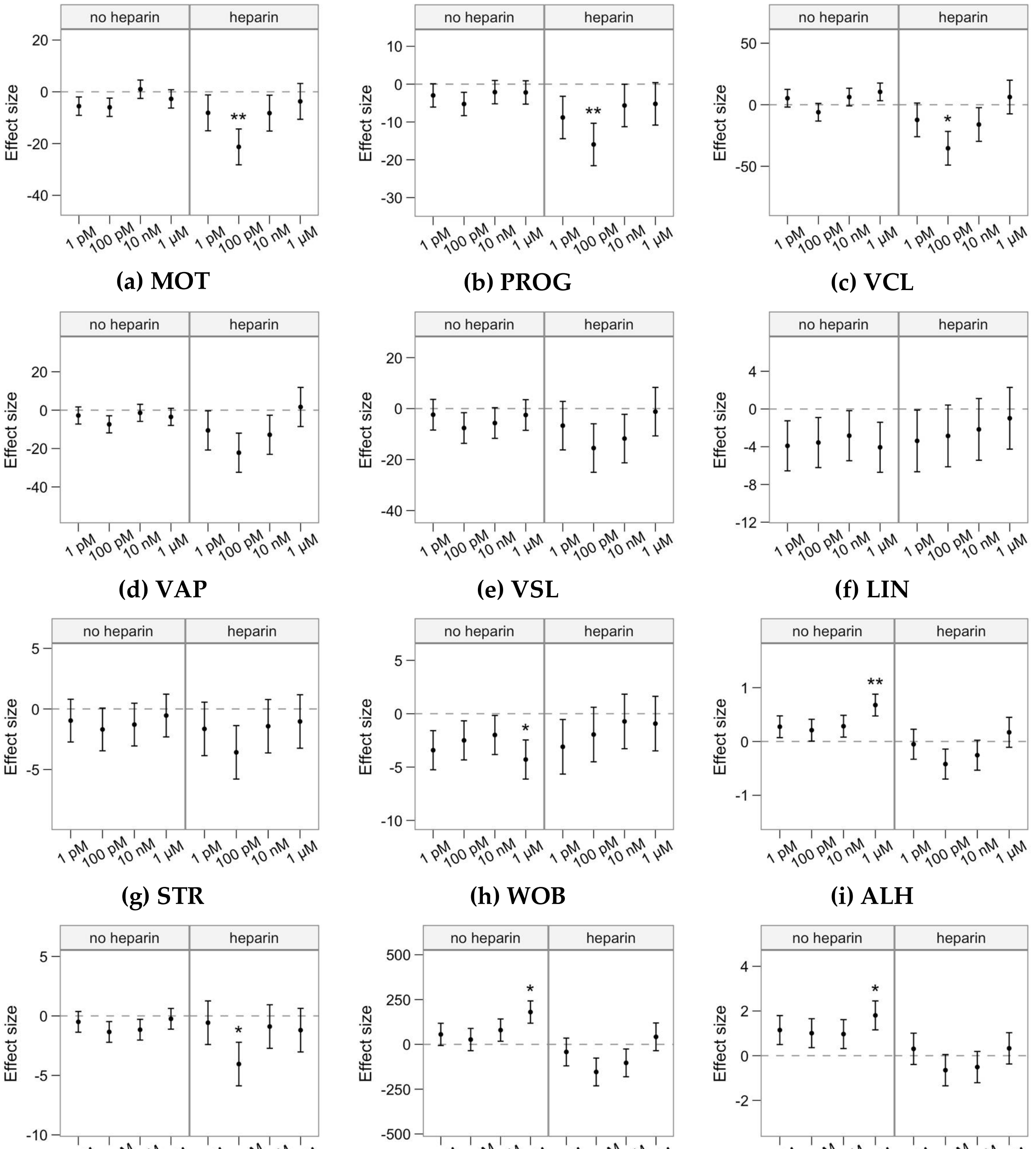

(a) MOT **(b) PROG** **(c) VCL**

(d) VAP **(e) VSL** **(f) LIN**

(g) STR **(h) WOB** **(i) ALH**

(j) BCF **(k) DNC** **(l) DNCm**

Figure S1. Effects of incubating bull spermatozoa with different melatonin concentrations on motility parameters (CASA analysis; Control, no melatonin, as reference). Plots show effect sizes (mean±SEM) for each concentration (0 as no effect) in the two in absence or presence of heparin. (a) Total molility. (b) Progressive motility. (c) Curvilinear velocity. (d) Average-path velocity. (e) Straight-path velocity. (f) Linearity. (g) Straightness. (h) Wobble. (i) Amplitude of the lateral displacement of the sperm head. (j) Frequency of the flagellar beat. (k) Dance. (l) Dance mean. Asterisks label effect sizes significantly different from 0 (* P<0.05, ** P<0.01, *** P<0.001).

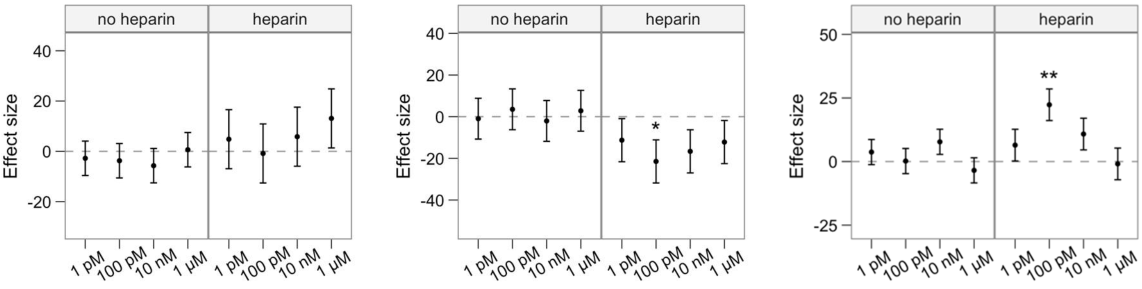

(a) Active **(b) Rapid** **(c) Slow**

Figure S2. Effects of incubating bull spermatozoa with different melatonin concentrations on motility subpopulations (Control, no melatonin, as reference). Plots show effect sizes (mean±SEM) for each concentration (0 as no effect) in the two in absence or presence of heparin. (a) "Active" spermatozoa, highest velocity and dance. (b) "Rapid" spermatozoa, high velocity and low dance. (c) "Slow" spermatozoa, lowest velocity and linearity. Asterisks label effect sizes significantly different from 0 (* P<0.05, ** P<0.01, *** P<0.001).

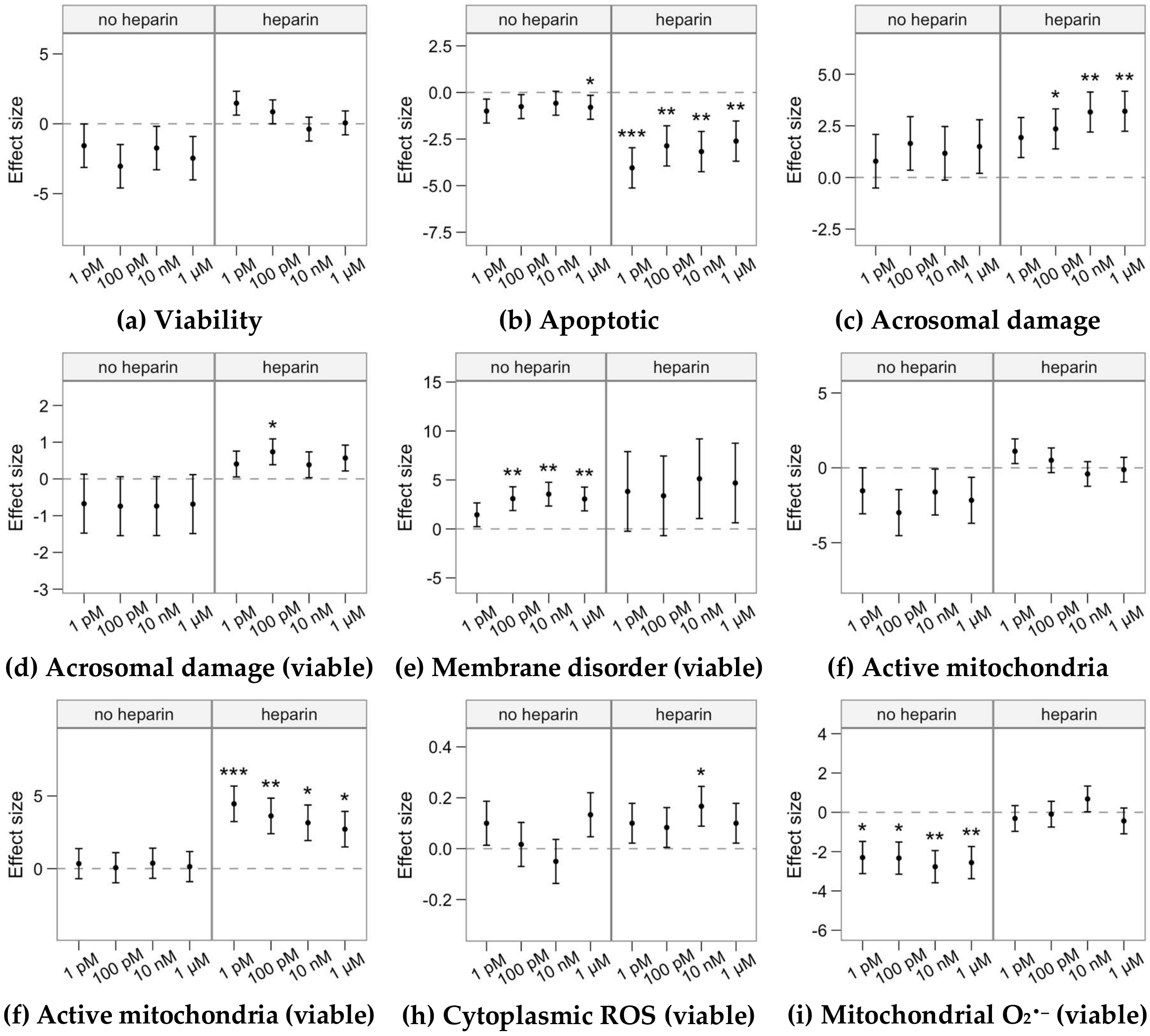

Figure S3. Effects of incubating bull spermatozoa with different melatonin concentrations on some physiological parameters (flow cytometry analysis; Control, no melatonin, as reference). Plots show effect sizes (mean±SEM) for each concentration (0 as no effect) in absence or presence of heparin. (a) Viable spermatozoa. (b) Apoptotic spermatozoa (YO-PRO-1^{+}). (c) Acrosome-reacted (damaged) spermatozoa. (d) Spermatozoa with damaged acrosome as ratico of viable spermatozoa. (e) Capacitated spermatozoa as with increased membrane disorder (M540+), as ratio of viable spermatozoa. (f) Spermatozoa with active mitochondria. (g) Spermatozoa with active mitochondria as ratio of viable spermatozoa. (h) Cytoplasmic ROS production of viable spermatozoa. (i) Spermatozoa with high mitochondrial superoxide production as ratio of viable spermatozoa. Asterisks label effect sizes significantly different from 0 (* $P<0.05$, ** $P<0.01$, *** $P<0.001$).

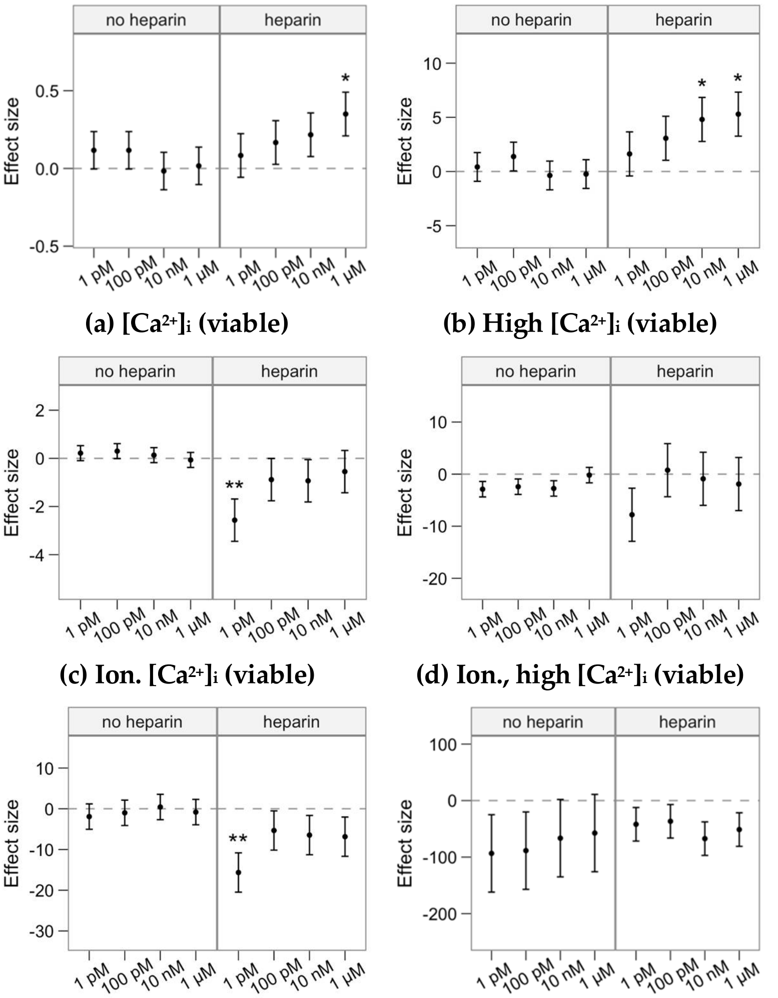

(a) $[Ca^{2+}]_i$ (viable)

(b) High $[Ca^{2+}]_i$ (viable)

(c) Ion. $[Ca^{2+}]_i$ (viable)

(d) Ion., high $[Ca^{2+}]_i$ (viable)

(e) Ion., ratio $[Ca^{2+}]_i$ (viable)

(f) Ion., ratio high $[Ca^{2+}]_i$ (viable)

Figure S4. Effects of incubating bull spermatozoa with different melatonin concentrations on intracellular calcium concentration (Control, no melatonin, as reference). Plots show effect sizes (mean±SEM) for each concentration (0 as no effect) in absence or presence of heparin. (a) Intracellular calcium concentration ($[Ca^{2+}]_i$, from mean fluorescence intensity of Fluo-4 in viable spermatozoa). (b) Spermatozoa with high $[Ca^{2+}]_i$ as ratio of viable spermatozoa. (c, d) Same parameters after ionophore treatment. (e, f) Same parameters as ratio of measurements after and before the ionophore treatment (ratio of change). Asterisks label effect sizes significantly different from 0 (* $P<0.05$, ** $P<0.01$, *** $P<0.001$).

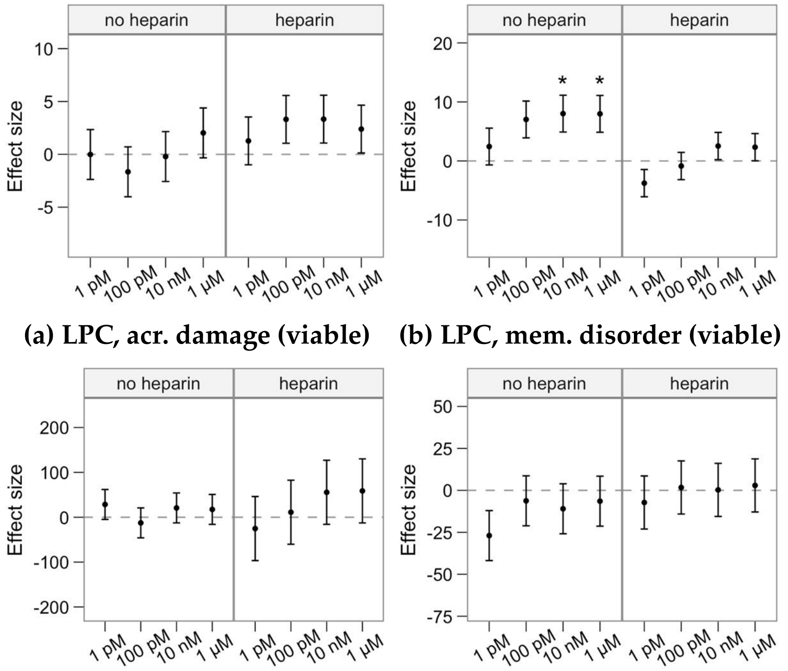

(a) LPC, acr. damage (viable) **(b) LPC, mem. disorder (viable)**

(c) LPC, acr. dam., ratio (viable) **(d) LPC, mem. dis., ratio (viable)**

Figure S5. Effects of incubating bull spermatozoa with different melatonin concentrations on the response to lysophosphatidilcholine challenge (Control, no melatonin, as reference). Plots show effect sizes (mean±SEM) for each concentration (0 as no effect) in absence or presence of heparin. (a) Spermatozoa with damaged acrosome as ratio of viable spermatozoa. (b) Capacitated spermatozoa as with increased membrane disorder (M540+), as ratio of viable spermatozoa. (c–d) Same parameters as ratio of measurements after and before the ionophore treatment (ratio of change). Asterisks label effect sizes significantly different from 0 (* $P<0.05$, ** $P<0.01$, *** $P<0.001$).

© 2020 by the authors. Submitted for possible open access publication under the terms and conditions of the Creative Commons Attribution (CC BY) license (http://creativecommons.org/licenses/by/4.0/).
